# Effective components screening and anti-myocardial infarction mechanism study of the Chinese medicine NSLF6 based on "system to system" mode

**DOI:** 10.1186/1479-5876-10-26

**Published:** 2012-02-08

**Authors:** Qiong-Lin Liang, Xiao-Ping Liang, Yi-Ming Wang, Yuan-Yuan Xie, Rong-Li Zhang, Xi Chen, Rong Gao, Yi-Jun Cheng, Jun Wu, Qing-Bo Xu, Qing-Zhong Xiao, Xue Li, Shu-Feng Lv, Xue-Mei Fan, Hong-Yang Zhang, Qing-Li Zhang, Guo-An Luo

**Affiliations:** 1 Department of Chemistry, Beijing Key Laboratory for Microanalytical Methods and Instrumentation, Tsinghua University, Beijing 100084, China; 2 School of Pharmacy, East China University of Science and Technology, Shanghai 200237, China; 3 Xiangxue Pharmaceutical, Guangzhou 510530, China; 4 Kings College London, London SE5 9NU, UK; 5 London School of Medicine and Dentistry, Queen Mary University of London, London SE5 9NU, UK; 6 Modern Research Center for Traditional Chinese Medicine, Tsinghua University, Beijing 100084, PR China

## Abstract

**Background:**

*Shuanglong *formula (SLF), a Chinese medicine composed of *panax ginseng *and *salvia miltiorrhiza *exhibited significant effect in the treatment of myocardial infarction (MI) in clinical. Because of the complex nature and lack of stringent quality control, it's difficult to explain the action mechanism of SLF.

**Method:**

In this study, we present a "system to system" (S2S) mode. Based on this mode, SLF was simplified successively through bioactivity-guided screening to achieve an optimized minimal phytochemical composition (new formula NSLF6) while maintaining its curative effect for MI.

**Results:**

Pharmacological test combining with the study of systems biology show that NSLF6 has activity for treatment MI through synergistic therapeutic efficacies between total ginsenosides and total salvianolic acids via promoting cardiac cell regeneration and myocardial angiogenesis, antagonistic myocardial cell oxidative damage.

**Conclusions:**

The present S2S mode may be an effective way for the discovery of new composite drugs from traditional medicines.

## Background

Dr. Zerhouni pointed out in NIH's Roadmap that translational medicine may evoke a great evolution of medicine in 21st century [1]. The conventional drug (western medicine) is screened based on single entity and its interaction with single target, representing as 'point to point' (P2P) mode. However, its R&D productivity has experienced decades of decline with the greatly increased cost and lengthened time [2]. Some of those problems of single-target-based screening may be overcome with the proposal of systems biology which believe that the body system is a holistic well-organized system composed of ordered networks including genes, proteins, metabolites, and so on. The network pharmacology based on the development of systems biology may represent an interaction mode of single (or multiple) point and biological system (point to system, P2S) [3]. Since Translational Medicine emphasizes on exploring the synergy and interaction of various networks and combining knowledge across disparate domains, interests are arouse if Translational Medicine will impact on Traditional Chinese Medicine (TCM) and catalyze the mixing of western medicine and eastern medicine.

TCM has not been fully accepted by mainstream medicine whereas it has a long history of clinical practice in China and beyond China. Besides of the complex nature of the formulae, as well as a lack of stringent quality control, the main obstacles of understand TCM may be attributed to its holistic treatment concept representing the interaction of drug system and human system which is quite different with the "P2P" mode of western medicine [4].

During its thousands of years' clinical practice, TCM formulas have being developed according to the routine of "Beside-Bench-Beside" which is also similar with the proposal of Translational Medicine. Many of the TCM formulas have a proven efficacy in clinical application. The pioneering work of Prof. Cheng's group from Yale University proved the efficacy of a TCM formula (PHY906) and interpreted its mechanism by modern pharmacological study [5], demonstrated the necessity and rationality of TCM's combination use to the international communities, and helped the communication of Chinese traditional medicine and modern medicine.

In the past study we have introduced approaches of Chemomics and systems biology to study the composition of a chemome (e.g. a TCM formula) and the correlation between its change and biological effect [6,7]. Prof. Sutherland from Brunel University commented that Chemomics represents an interesting synthesis of both Eastern and Western culture and provide a new "omics" approach to develop "modernized composite medicine" (MCM), where "the phytochemical composition of a herbal formula with demonstrated clinical efficacy is regarded as a global chemome, which can be simplified successively through bioactivity-guided screening to achieve an optimized chemomome with minimal phytochemical composition for further drug development, while maintaining its curative effect for a specific disease" [8].

**Figure 1:**
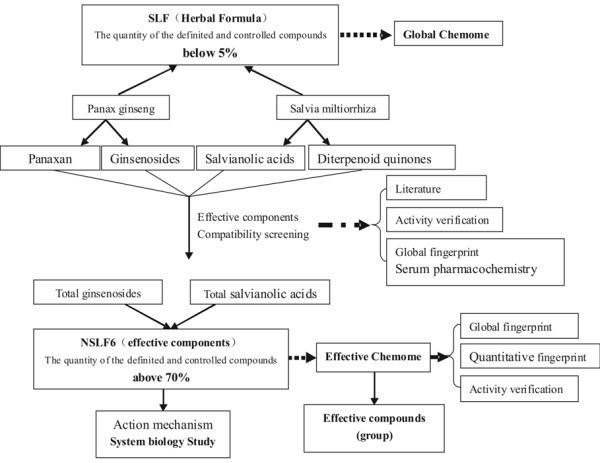
**Screening and discovery of NSLF6 based on "system to system" mode**.

Here we present a mode of "system to system" (S2S) by integrating Chemomics and systems biology which is so called Integrative System Biology approach to study the interaction of drug system and biological system (Additional file 1: Figure S1). Different from conventional lead compound screening based on the selected target (P2P mode), the presented S2S methodology is advantaged for TCM formula with proven clinical efficacy, characterizing the chemical composition and their relationship of the TCM drug system by means of Chemomics, characterizing the response of the biological system by means of Systems Biology, providing a comprehensive approach for understanding the interaction of both systems. As a demonstrative study, the development of a new drug (NSLF6) for therapy of myocardial infarction (MI) from TCM *shuanglong *formula (SLF) was presented here. The Chinese medicine SLF, a combination of *panax ginseng *(PG) and *salvia miltiorrhiza *(SM) at a ratio of 7:3, has been used for the clinical treatment of cardiovascular diseases such as myocardial infarction (MI) and angina pectoris over ten years by Professor Lianda Li, Xiyuan Hospital of China Academy of Chinese Medical Sciences. Former studies based on MI models of rats, pigs, and dogs showed that SLF alone or combined with mesenchymal cell transplantation could reduce myocardial infarct area and the degree of myocardial injuries, improve cardiovascular function, and increase myocardial blood and myocardial capillary density [9-11]. Nevertheless, as similar as the most traditional medicines, the poorness and difficulty in quality control and pharmacological interpretation was one of the bottlenecks for further development of new drugs. Therefore, it is necessary to simplify it successively through bioactivity-guided screening to achieve an optimized minimal phytochemical composition and interpret its action mechanism clearer. The roadmap of the study is shown in Figure 1.

## Methods

*Panax ginseng*, *Salvia miltiorrhiza*, SLF and NSLF were provided by Guangzhou Xiangxue Pharmaceutical Co., Ltd. The detailed descriptions for the studies of global chemome fingerprint and quantitative fingerprint analysis together with serum pharmacochemistry were given in the Supplementary Material.

### Methods of MI rat models

The animal experiments were operated in accordance with the standards established by the Guide for the Care and Use of Laboratory Animals of Beijing city and approved by the local ethics committee (IACUC#: 2010-LuoGA-SMI). Methods of isoproterenol (ISO)-induced MI rat model and coronary artery ligation-induced MI rat model were the same as before [12,13].

### Metabolomic study methods

Method parameters of Metabolomic study of ISO-induced MI rat serum and coronary artery ligation-induced MI rat urine were the same as before [12,13].

### Cell culture

Human Umbilical Vein Endothelial Cells (HUVECs) were routinely cultured in Medium RPMI1640 supplemented with 10% fetal bovine serum (FBS), 100 μg/ml penicillin/streptomycin. These cells were cultured in 5% CO_2 _at 37°C and the media were replaced at 2-day intervals.

### HUVECs proliferation assay

HUVECs were grown to 70% confluence in 96-well plates and cultured with indicated concentration of GS, TSA and NSLF6. Cell proliferation was both measured using MTT assay and direct counting. Each experiment was carried out with five replicates per treatment and was independently repeated more than three times.

### Migration assay

Effect of NSLF on HUVECs migration was studied using BD Chambers with Polycarbonate filters (8.0 μm pore). Suspended cells (5 × 10^4^) were placed on the filter in RPMI1640 0.1% bovine serum albumin (BSA) containing indicated concentrations of NSLF. In the lower chamber RPMI1640 supplemented with 5% FBS was added. After incubated 3 h at 37°C, the filter was removed, and the upper side of the filter containing the nonmigrated cells was wiped and rinsed. The filters were fixed with 4% paraformaldehyde and stained with 4',6-diamidino-2-phenylindole (DAPI). Migration was quantified by counting cells. All groups were studied in triplicate.

### cDNA microarray

The cDNA microarray hybridization was performed as previously described [14].

### Real-time PCR

Total RNA was extracted from the cardiac tissue using TRIZOL reagent (Invitrogen) and further purified with RNeasy affinity column (MN). First-strand cDNA was synthesized using First strand cDNA synthesis Kit (Fermentas). Primers of Arnt1, Nppa and GAPDH were designed by primer 3.0 (the information of primers was shown in Additional file 1: Table S1). Each sample was measured in triplicate. Cycle threshold (Ct) value of each sample was obtained and 2^-ΔΔCt^. Relative quantification was used to calculate the gene expression. Each quantitative PCR was preformed twice.

### Capillary-like structure formation

HUVECs (10^4 ^cells/well) were cultured on growth factor reduced-Matrigel (BD Biosciences) coated 24 plates in RPMI1640 0.1% BSA containing 10-40 μg/ml NSLF6 or vehicle. When cultured on matrigel, cells assemble into capillary-like structures. After incubated 18 h, the cord-like structures were observed by an inverted microscope.

## Results

### Screening of effective components in SLF

We have reported a urine metabolomic study combined with pharmacological tests based on coronary artery ligation-induced MI rat model and concluded that SLF (in its original form) exhibited appealing therapeutic efficacies on MI [10].

**Figure 2:**
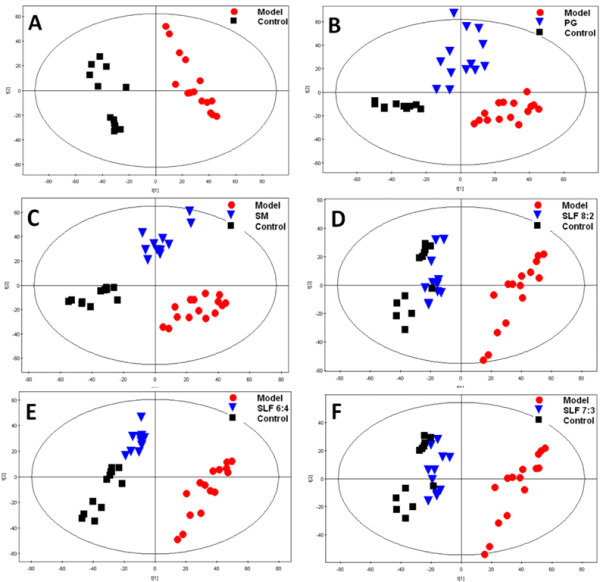
**PCA scores plots of ISO-induced MI rat serum**. The rats were administrated with test drugs for 7 days at the same doses of 5 g/kg·w·d and twice injections of ISO on days 6 and 7 to induce MI model. The samples were collected on the day 7. Serum samples were analyzed using UPLC/TOF-MS and the data were processed by PCA in the software SIMCA-P package. SLF 8:2, 7:3, and 6:4 represent the ratios of PG to SM. Details of the experimental procedures are given in Materials and Methods.

A serum metabolomic study combined with pharmacological tests based on ISO-induced MI rat model was also conducted here to validate the efficacy and optimize the ratio of herbal combination of SLF. As shown in Figure 2A, the metabolic state of model group was far away from the normal control group, indicating the success of ischemia model. Both PG group (Figure 2B) and SM group (Figure 2C) were separate with the model group but away from the control group. Among the groups of control, model, and different combinations of PG and SM (8:2, 7:3, and 6:4), the group of 7:3 (as original ratio as SLF) and 8:2 were the nearest to the control group and obvious away from the model group, as shown in Figure 2D-F. Combined with pharmacological tests such as electrocardiogram (ECG) changes, activity of enzymes (CK, LDH, SOD, and MDA), and myocardial infarction area (Additional file 1: Figure S2, Additional file 1: Table S2-S4), we can conclude that the combination-based treatment groups were better than the single herb (PG or SM) groups and the combination of PG and SM at a ratio of 7:3 and 8:2 exhibited the best therapeutic efficacies on MI rats. Since there is little significant difference between the groups of 7:3 and 8:2, the original combination namely 7:3 has been recommended for further development considering its stronger basis of the clinical practice.

**Figure 3:**
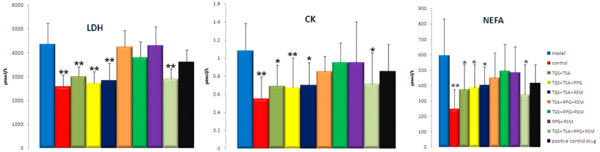
**Comparison of LDH, CK, and NEFA activity in serum of ISO-induced MI rats administrated different combinations**. TGS: total ginsenosides, RPG: the remainder of PG (mainly containing panaxan, TGS removed), TSA: total salvianolic acids, RSM: the remainder of SM (mainly containing diterpenoid quinones, TSA removed). *P < 0.05, **P < 0.01 vs model group. Positive control drug: compound danshen pills. Details of the experimental procedures are given in Materials and Methods.

The serum-chemical and chemomics research results indicated that ginsenosides and salvianolic acids might be the effective components of SLF for MI (Additional file 1: Table S5). To further verify the above deduction, the two herbs (PG and SM) in SLF were divided into four parts: total ginsenosides (TGS), the remainder of PG (RPG, mainly containing panaxan, TGS removed), total salvianolic acids (TSA), and the remainder of SM (RSM, mainly containing diterpenoid quinones, TSA removed). After cross combinations (equal to the ratio of 7:3 of PG to SM), their efficacies were evaluated using an ISO-induced MI rat model. As shown in Figure 3, a significant reduction (*P *< 0.05) in CK, LDH, and NEFA levels was observed in rats treated with the TGS + TSA, TGS + TSA + RPG, TGS + TSA + RSM, and TGS + TSA + RPG + RSM (the original constitution of SLF) compared with the model group. There were not statistically significant differences (*P *> 0.05) in the TSA + RPG + RSM, TGS + RPG + RSM, and RPG + RSM groups compared with the model group. The activities of TGS + TSA were almost the same as the TGS + TSA + RPG + RSM. Besides, the results were confirmed by the ECG changes and metabolomic studies (Additional file 1: Figure S3, Additional file 1: Table S6).

On the one hand, the combination TGS + TSA exhibited equal pharmacological activities with the original SLF and the removal of the rest parts (RPG, RSM) had no significant effect on activities. On the other hand, absence of either/both of ginsenosides and salvianolic acids led to losing of anti-ischemic efficacy. Therefore, we took the components of ginsenosides and salvianolic acids as the indispensable effective components and equivalence contributed to the anti-MI efficacy of SLF. As a result, we obtained a new prescription with simplified compositions and maintaining efficacy, combination of TGS and TSA as the equal ratio of which present in the original formula SLF, named as "NSLF6".

### Study on chemistry and activity confirmation of NSLF6

The global chromatographic fingerprint of the NSLF6 was established by UPLC/TOF-MS. A total of 20 compounds in NSLF6 were separated and identified, among of which there were 12 ginsenosides and 8 salvianolic acids. The quantitative fingerprints of total ginsenosides and total salvianolic acids were established by HPLC-DAD. As shown in Additional file 1: Table S7 and Additional file 1: Figure S4, the total contents of quantitative compounds such as ginsenosides Rb_1_, Rg_1_, Ro, salvianolic acid B, and lithospermic acid accounted for 74.4% of total solids. Thus, most of the constituents in NSLF6 have been identified and quantifiable so that we have established a stringent quality control, which provided the chemical basis for unveiling the pharmacological mechanism. Additionally, the comparative study of components in plasma of rats before and after administration of NSLF6 as well as NSLF6 was conducted by UPLC/TOF-MS. The results showed that 5 salvianolic acids including salvianolic acid B, rosmarinic acid, propanoid acid, salvianolic E, and lithospermic acid, and 7 ginsenosides concluding ginsenoside Rb_1_, Rb_2_, Re, Rd, Rf, M-Rb_1_, and M-Rb_1 _from NSLF6 were detected in plasma.

**Figure 4:**
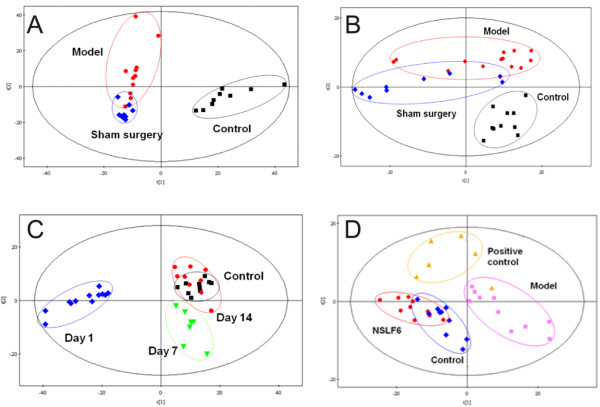
**PLS-DA scores plots of rat urine data**. (A) Dynamic mean-centered PLS-DA score plot of rat urine data of model, sham surgery, and control groups on day 1. (B) Dynamic mean-centered PLS-DA score plot of rat urine data of model, sham surgery, and control groups on day 14. (C) Dynamic mean-centered PLS-DA score plot of rat urine data of NSLF6 group on days 0, 1, 7, and 14. (D) Dynamic mean-centered PLS-DA score plot of rat urine data of NSLF6 and positive control drug group on day 14. Positive control drug: diltiazem hydrochloride. Details of the experimental procedures are given in Materials and Methods.

The activities of NSLF6 were evaluated by metabolomics and pharmacological tests using MI rats induced by coronary artery ligation. As shown in Figure 4A, the endogenous metabolites in the model group were obviously different from that of the normal control group, indicating the success of myocardial ischemia model. After two weeks' survival without medication (Figure 4B), the trajectory of the model group was far away from the position of the normal control, which excluded the suspicion of self-cure of the MI rats during the experiment. As shown in Figure 4C, the metabolic state of the NSLF6 group was far away from the position of normal control on the first day after coronary artery ligation while the trajectory direction gradually moved to the normal control during two weeks' medication, indicating the recovery of the disturbed metabolism state by NSLF6. In Figure 4D, both the NSLF6 and positive control groups moved to the normal control group, and the NSLF6 group exhibited better performance in the recovery of metabolism than the positive control group. The results confirmed that the NSLF6 had a proven efficacy in coronary artery ligation induced MI rats. In addition, the pharmacological results (Additional file 1: Table S8-S9, Additional file 1: Figure S5) showed that the NSLF6 could reduce the serum enzyme activity, diminish the area of myocardial infarction, and increase the myocardial capillary density [15].

### NSLF6 promotes Human Umbilical Vein Endothelial cells (HUVECs) proliferation, migration and angiogenesis

NSLF6 could significantly reduce myocardial infarction area, and one of the mechanisms might be related to promote angiogenesis and antagonize cardiomyocytes oxidative damage.

**Figure 5:**
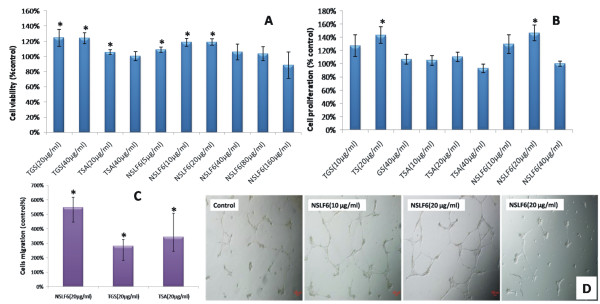
**Effects of NSLF6 on HUVECs proliferation, migration, and angiogenesis**. (A) & (B) Cells were incubated for 24 h with GS, TSA and NSLF6. Cell proliferation was evaluated by MTT assay (A) and cell counting (B). (C) Cell migration was examined by transwell chambers with polycarbonate filters (8.0 μm pore) after incubation for 3 h. (D) Cells were seeded onto matrigel in 24-well plates. After incubated with NSLF6 for 18 h, tube formation was recorded photographically (40×). Results are expressed as percentage of control.*P < 0.05 versus control. *n *= 5. Details of the experimental procedures are given in Supplementary Materials.

Among NSLF6, TSA and TGS, NSLF6 (20 μg/ml) exhibited the best activity in promoting cells proliferation (Figure 5A-B). The determination of cell migration showed that NSLF6, TSA and TGS significantly enhanced the ability of cell migration (Figure 5C), and NSLF6 was optimal with migration rate increased 500%. To evaluate the simulative effect on angiogenesis, we treated HUVECs with NSLF6 (10, 20 and 40 μg/ml) for 18 h and examined their ability to form tubular formation. 20 μg/ml NSLF6 exhibited maximum promotion of tubule formation (Figure 5D). The results showed that NSLF6 had the effect on promoting HUVECs proliferation, migration and angiogenesis.

### NSLF6 antagonizes cardiomyocytes oxidative damage

**Figure 6:**
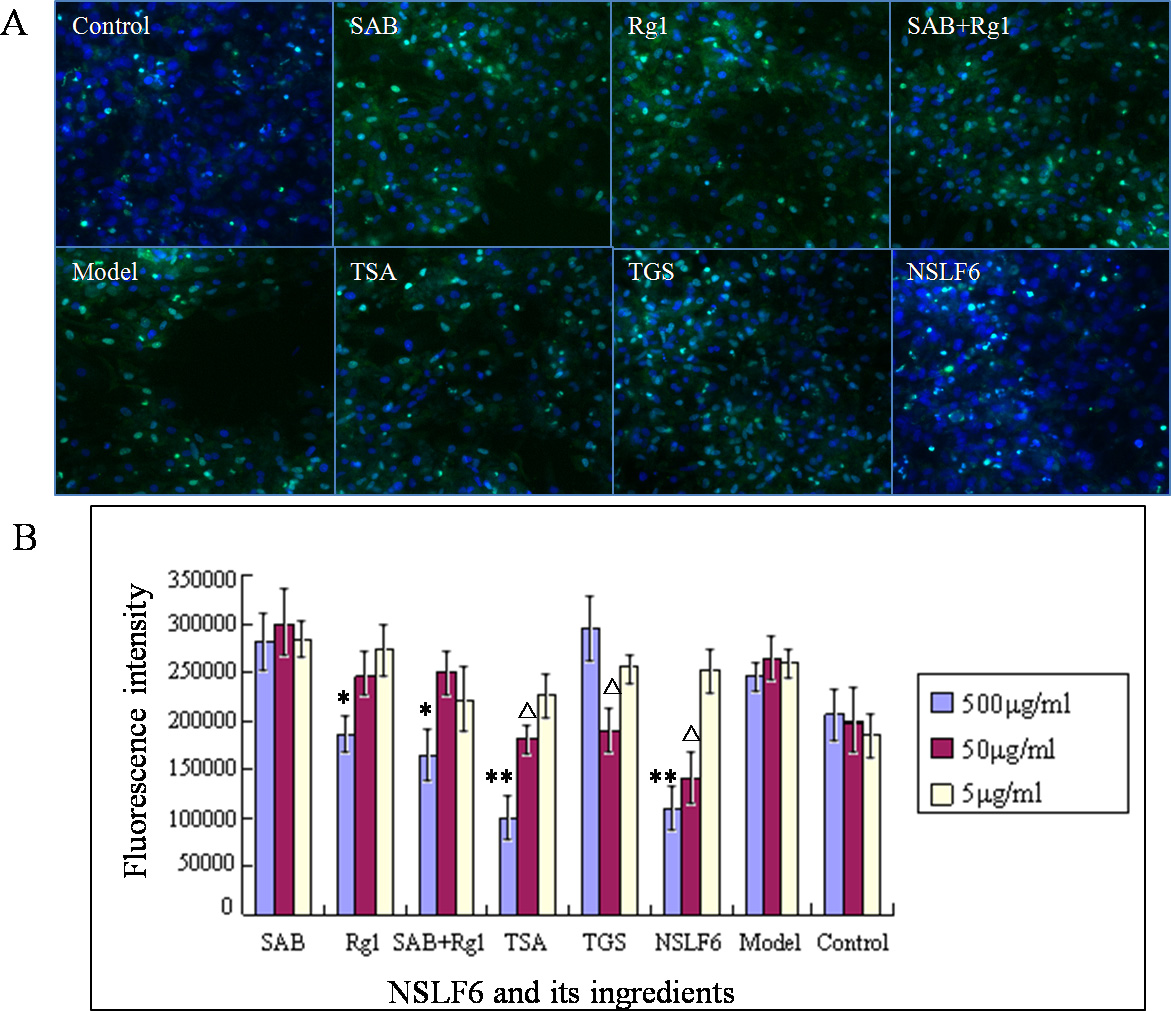
**Effect of NSLF6 and its effective ingredients on the membrane permeability of cardiomyocytes by H_2_O_2_**. (A) Fluorescence expressions of membrane permeability of the damaged cardiomyocytes stimulated by NSLF6 and its effective ingredients. Green fluorescent shows the membrane permeability, blue fluorescence shows nucleus stained by DAPI. (B) Comparison of NSLF6 and its effective ingredients to the membrane permeability of cardiomyocytes by H_2_O_2_. Details of the experimental procedures are given in Supplementary Materials.

The model of cardiomyocytes damaged by H_2_O_2 _was used to study the protective effect of NSLF6 and its effective ingredients. After cardiomyocytes were co-cultured with these drugs for 48 h, 10% H_2_O_2_--PBS solution was added to the medium, cultured for 2 h, thereafter high content screening (HCS) system was used to analyze the health degree of cardiomyocytes [16]. The results indicated that the mitochondrial membrane potential of those cardiomyocytes treated with NSLF6 and its effective ingredients was higher, the membrane permeability was lower and the nuclear was integrity, compared with the model. These results suggested that NSLF6 had the effect of antagonizing the damage of cardiomyocytes induced by H_2_O_2 _(Figure 6, Additional file 1: Figure S6). TSA and one of its major constituents, salvianolic acid B (SAB) could both significantly improve the mitochondrial membrane potential of the damaged cardiomyocytes (Figure 6A-B), TGS significantly reduced the membrane permeability, and NSLF6 had the optimized effect on the maintenance of nuclear integrity (Additional file 1: Figure S6). Therefore, all of NSLF6 and its effective ingredients could recover the rat cardiomyocytes damaged by H_2_O_2_. In summary, combined the results from three tests of mitochondrial membrane potential, the membrane permeability and the nuclear integrity, NSLF6 had the best effects and the optimal concentration was 10^-4 ^mol/l. In addition, the investigation on the model of cardiomyocytes damaged by Hypoxia/Reoxygenation (H/R) also confirmed that NSLF6 had the protective effect on damaged cardiomyocytes, and such a pharmacological action was mainly contributed by TGS (Additional file 1: Table S10).

### Study of NSLF6 based on Systems biology

**Figure 7:**
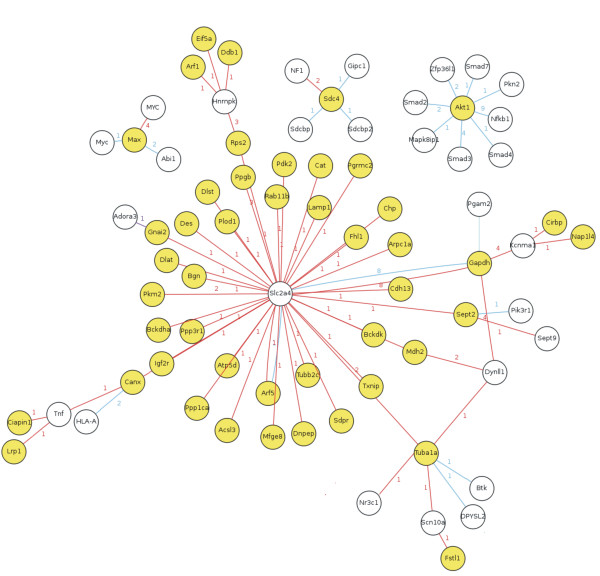
**The protein interaction network related to the anti-MI effect of NSLF6**. Yellow means the genes encoding the protein contained the differentially expressed genes in this experiment. Details of the experimental procedures are given in Supplementary Materials.

Taking the coronary artery ligation induced MI rats model, using cDNA microarray, the myocardial gene expression profile was determined to screen the differentially expressed gene induced by administrating NSLF6. As a result, 476 genes displayed significant difference (filter: the change ratio ≥ 2, n ≥ 4. Additional file 1: Figure S7 showed the results of cluster analysis and PCR verification). After administrated NSLF6, the expression of most differentially expressed genes was closed to the SHAM group. By further analysis of the differentially expressed gene, the relational protein interaction network was preliminarily obtained (Figure 7). The glucose transporter 4(GLUT4/slc2a4) was in the center of interaction network. It suggested that GLUT4 played a key role in the process of NSLF6 administration. Glucose is the main material for providing energy, and GLUT4 is the carrier of glucose and play an important role in glucose uptake. N-Begum et al [17] reported that increased intracellular calcium concentration could activate the phosphorylation of GLUT4 to inhibit its intrinsic activity for glucose transport. Moreover, gene functional analysis found that the differentially expressed genes were mainly related to the pathways about energy metabolism, ion binding, MAPK, and VEGF. These results suggested that NSLF6 might mainly regulate the internal ion concentration and energy metabolism to display the efficacy. In addition, protein kinase B (Akt/PKB) also involved in NSLF6 treatment. And the PI3K/Akt pathway had noteworthy effect in the course of MI treatment [18,19].

Using the same MI model as above, 24 of 26 differential endogenous metabolites in urine were identified using high resolution mass spectrometry, involved in metabolic processes related to myocardial energy metabolism (myocardial energy metabolism, the citric acid cycle, and amino acid metabolism) (Additional file 1: Table S11). Among them, the levels of glucaric acid, uridine, D-Glucuronic acid 1-phosphate, deoxyadenosine monophosphate, 6-Phosphogluconic acid, and ceramide were observed significantly increased and the others were significantly decreased in model group compared with the normal control group. The observation of the differential endogenous metabolites moved to nearly normal levels in NSLF6 group indicated that NSLF6 may exert therapeutic efficacies on MI by regulating these metabolomic networks. For the study on SLF, we have indentified 17 potential biomarkers related to the effect of SLF [13], and we find that 13 of them are also contributed to the effect of NSLF6 and exhibit the consistent variation trend. Furthermore, the 14 differential endogenous metabolites in serum obtained from MI rats induced by ISO had the same trends as the urine metabolites (Additional file 1: Table S12).

Furthermore, the differentially expressed genes and proteins were screened through comparative genomics and comparative proteomics during the differentiation from BMMSCs into cardiomyocyte-like cells induced by NSLF6. 180 differentially expressed genes were obtained, in which 10 genes (fold change ≥ 10) were confirmed by Real-Time PCR, and obtained the functional relationship (Additional file 1: Figure S8). The differentially expressed proteins were obtained between control group and the differentiation group, and the relative signaling pathway network was obtained (Additional file 1: Figure S9). These results suggested that the MAPK, calcium binding, Rho and Wnt pathways played important roles during the BMMSCs differentiation [20]. And NSLF6-induced differentiation was mainly attributed to the MAPK (eEF-2, Actb and Nme2), calcium binding (S100 calcium binding protein) and the Rho (Vim) signaling pathways (Additional file 1: Table S13) [21].

DiscussionTraditional Chinese Compound Medicine (TCCM) is known as multi-component drug capable of targeting multiple sites through multiple mechanism of action at multiple pathological steps. But these actions represent integral regulation other than the sum of individual target. Therefore, chemomics-integrated systems biology (integrative systems biology, ISB), a holistic methodology, was developed for the study of interaction between external intervention system (TCM) and biological response system (human system) based on S2S mode. The Traditional Chinese Formula is a compatible combination of raw herbs, in which indispensable components co-exist with ineffective components. For further drug development, it is necessary to remove the ineffective components while maintaining its curative effect for a specific disease. Consequently, we developed a S2S mode by which a complex formula can be simplified successively through bioactivity-guided screening to achieve a minimal effective composition with definite constituents and controllable quality, so that its mechanism can also be interpreted. In the present study, TGS and TSA were screened from SLF according to the studies of chemomics, serum pharmacochemistry, metabolomics and activity evaluation. NSLF6, the simplified prescription with components combination of TSA and TGS was obtained, and the systems study on the action mechanism of NSLF6 was conducted.

According pharmacological studies based on the MI rat model induced by ISO or the coronary artery ligation, NSLF6 can treat MI via several mechanisms such as promoting cardiomyocytes renewal, promoting angiogenesis and antagonizing cardiomyocytes oxidative damage.

It has been reported that there are stem cells existing in the heart of adult [21]. Whether NSLF6 has the effect of promoting the differentiation of stem cells into cardiomyocytes to treat myocardial infarction? The *in vivo *experiments showed that SLF with bone marrow mononuclear cells (BMMCs) auto-transplanted into myocardial infarction model in swines could significantly improve the survival, differentiation and amplification of the transplanted cells in myocardial tissue, generate a raft of new cardiomyocytes and microvascular, and eventually promote lesions recovery, compared with BMMCs group [13]. The above *in vitro *study proved that SAB, the effective ingredient of NSLF6, effectively induced the differentiation of mESCs into cardiomyocytes with physiological beating frequency. Except Vc and retinoic acid, there has been few drug reported to have the inducing effect on ESC differentiating into cardiomyocytes. Thus, NSLF6 has a good potential and specific advantage in the treatment of MI. The study of genomic and metabolomic above displayed the NSLF6 activity of regulating energy metabolism, transcription factors and oxide reductase activity, which was reported to be involved in the differentiation of mESCs into cardiomyocytes [22].

Partial angiogenesis and remodeling after MI is an important part of Cardiac Tissue Repair. Therapeutic angiogenesis is the clinical use of methods to enhance or promote the development of collateral blood vessels in ischemic tissue, and vividly described as "self-medication for heart bypass". The study of genomic and metabolomic above displayed the NSLF6 activity of regulating the energy metabolism, ion binding, MAPK and VEGF pathway which was reported to be the most Specific and powerful regulators to promote angiogenesis [23]. Therefore, we considered that NSLF6 could promote angiogenesis through adjusting VEGF pathway, and confirmed the promoting angiogenesis of NSLF6 on HUVECs in vitro. Ginsenoside Rg_1_, one of ingredients in NSLF6, could stimulate the expression of VEGF to enhance the angiogenesis in previous report [24].

The H_2_O_2 _oxidative damage model of myocardial cells was widely used in the study of ischemia/reperfusion injury and ischemic preconditioning. It presents as the destruction of nuclear integrity and membrane permeability, increased mitochondrial membrane potential, oxygen free radicals, myocardial damage induced increase of enzyme activity, cell apoptosis, calcium overload and so on [25]. The activities of MDA [26], SOD [27] and LDH [28] were closely related to the myocardial oxidative damage. The study of cardiomyocytes model based on HCS system and MI rats model based on pharmacological test suggested that NSLF6 could effectively protect cardiomyocytes via several mechanisms that act simultaneously, such as decreasing lipid peroxidation, improving the myocardial cell' ability of oxygen free radicals, and resisting apotosis.

## Conclusion

Based on the integrative system biology mode, the traditional Chinese medicine SLF, as a clinical effective medicine, was developed to a new formula--NSLF6 with comparatively clear phytochemical composition, comparatively clear mechanism, and controllable quality. The stringent quality control system has been established using combination of fingerprint and multi-component determination. NSLF6 was proved to be effective on MI by pharmacological test (four levels of animal, tissue, cell, and molecular) combining with the study of gene, protein, and metabolite networks. The results revealed that SLF produced efficacy against MI through synergistic therapeutic efficacies between TGS and TSA. The present S2S mode may be an effective way for the discovery of new compound medicines.

## Abbreviations

SLF: *Shuanglong *formula; MI: myocardial infarction; P2P: point to point; P2S: point to system; S2S: system to system; TCM: Traditional Chinese Medicine; PG: panax ginseng; SM: salvia miltiorrhiza; ECG: electrocardiogram; TGS: total ginsenosides; RPG: the remainder of PG; RSM: the remainder of SM; HUVECs; Human Umbilical Vein Endothelial cells; HCS: high content screening; H/R: Hypoxia/Reoxygenation; TCCM: Traditional Chinese Compound Medicine; BMMCs: bone marrow mononuclear cells; ISO: isoproterenol; LDH: lactate dehydrogenase; CK: creatines kinases; NEFA: nonesterified fatty acid; MDA: malondialdehyde.

## Competing interests

The authors declare that they have no competing interests.

## Authors' contributions

Q-LL, X-PL, and H-YZ performed the experiments (metabonomics). Y-YX, Y-JC and JW performed the experiments (TCM research). Q-BX, Q-ZX, XL, S-FL and X-MF performed the experiments (genomics & proteomics). XC, RG, and R-LZ performed the experiments (animal). Q-LL, X-PL, Y-YX, S-FL, X-MF and Q-LZ participated in writing. G-AL designed and guided the whole experiments and wrote the paper. All authors read and approved the final manuscript.

## Supplementary Material

Additional file 1 **Supplementary materials**. Click here for file

## References

[B1] Zerhouni EA Translational and clinical science-time for a new vision N Engl J Med 2005 353 1621 1623 10.1056/NEJMsb053723 16221788

[B2] Booth B Zemmel R Prospects for productivity Nat Rev Drug Discov 2004 3 451 456 10.1038/nrd1384 15136792

[B3] Hopkins AL Network pharmacology: the next paradigm in drug discovery Nat Chem Biol 2008 11 682 690 10.1038/nchembio.118 18936753

[B4] Luo GA Liang QL Wang YM Liu QF Li X A perspective on the development of TCM systems biology Chin J Nat Med 2009 7 242 248

[B5] Lam W Bussom S Guan FL Jiang ZL Zhang W Gullen EA Liu SH Cheng YC The four-herb Chinese medicine PHY906 reduces chemotherapy-induced gastrointestinal toxicity Sci Transl Med 2010 2 1 8 10.1126/scitranslmed.3001270 20720216

[B6] Luo GA Liang QL Zhang RL Wang YM Liu QF Hu P Study of chemomics and prescription of traditional chinese medicine--And an analysis of material foundation of compound prescription qingkailing World Sci Tech/Moder Trad Chin Materi Med 2006 8 6 15

[B7] Luo GA Liang QL Liu QF Zhang RL Yang HH Li X Wang YM Jia W Zhang WD Zhang C Li YK Chemomics-integrated global systems biology: a holistic methodology of study on compatibility and mechanism of formulas in traditional chinese medicine World Sci Tech/Moder Trad Chin Materi Med 2007 9 11 15

[B8] Sutherland IA Fisher D Role of counter-current chromatography in the modernisation of Chinese herbal medicines J Chromatogr A 2009 1216 740 753 10.1016/j.chroma.2008.11.095 19108842

[B9] Li LD Zhang RL Liu CY Ning KY Li YK Therapeutic effects of *shuanglon *prescription for myocardial infarction in rats Trad Chin Drug Res & Clin Pharm 2004 15 149 151

[B10] Li LD Zhang RL Liu CY Ning KY Li YK Feng XQ He J Effects of shuanglong prescription combining with autologous bone marrow mononuclear cells transcatheter transplantation on myocardial infarction of Chinese miniature pig Med World 2004 3 20 23

[B11] Li LD Li YK Ning KY Zhang RL He J Effects of *shuanglon *prescription on heart hemodynamics and myocardial oxygen consumption in dogs Trad Chin Drug Res & Clin Pharmacol 2003 14 393 395

[B12] Zhang HY Chen X Hu P Liang QL Liang XP Wang YM Luo GA Metabolomic profiling of rat serum associated with isoproterenol-induced myocardial infarction using ultra-performance liquid chromatography/time-of-flight mass spectrometry and multivariate analysis Talanta 2009 79 254 259 10.1016/j.talanta.2009.03.045 19559874

[B13] Liang XP Chen X Liang QL Zhang HY Hu P Wang YM Luo GA Metabonomic study of Chinese medicine shuanglong formula as an effective treatment for myocardial infarction in rats J Proteome Res 2010 10 790 799 21090666 10.1021/pr1009299

[B14] Shi M Fan XM Li X Wang Z Wang YM Luo GA Gene expression studies of melamine-related renal toxicity based on cDNA microarray Chem J Chin Univer 2010 3 684 689

[B15] Gao R Liu QF Li YK Shi J Wang YM Luo GA Therapeutic effects of shuanglong prescription for experimental myocardial infarction Chin Trand Patent Med 2010 32 746 749

[B16] Abraham VC Towne DL Waring JF Warrior U Burns DJ Toxicity potential in humans application of a High-Content multiparameter cytotoxicity assay to prioritize compounds based on toxicity potential in humans J Biomol Screen 2008 13 527 537 10.1177/1087057108318428 18566484

[B17] Begum N Leitner W Reusch JE Sussman KE Draznin B GLUT-4 phosphorylation and its intrinsic activity. Mechanism of Ca(^2+^)-induced inhibition of insulin-stimulated glucose transport J Biol Chem 1993 268 3352 3356 8381427

[B18] Wang YH Wang SP Wier WG Zhang QJ Jiang HK Li QX Chen SF Tian ZJ Exercise improves the dilatation function of mesenteric arteries in postmyocardial infarction rats via a PI3K/Akt/eNOS pathway-mediated mechanism Am J Physiol Heart Circ Physiol 2010 299 H2097 H2106 10.1152/ajpheart.00701.2010 20935150

[B19] Liu YB Yu B Li SF Fan Y Han W Yu JB Wang Z Li XL Sun LP Yang SS Mechanisms mediating the cardioprotective effects of rapamycin in ischaemia-reperfusion injury Clin Exp Pharmacol Physiol 2011 38 77 83 10.1111/j.1440-1681.2010.05467.x 21126261

[B20] Fan XM Li X Lv SF Wang YM Zhao YF Luo GA Comparative proteomics research on rat MSCs differentiation induced by *shuanglon *formula J Ethnopharmacol 2010 131 575 580 10.1016/j.jep.2010.07.036 20659544

[B21] Urbanek K Cesselli D Rota M Nascimbene A Hosoda T Bearzi C Boni A Bolli R Kajstura J Anversa P Leri A Stem cell niches in the adult mouse heart PNAS 2006 103 9226 9231 10.1073/pnas.0600635103 16754876 PMC1474010

[B22] Chung S Arrell DK Faustino RS Terzic A Dzeja PP Glycolytic network restructuring integral to the energetics of embryonic stem cell cardiac differentiation J Mol Cell Cardiol 2010 48 725 734 10.1016/j.yjmcc.2009.12.014 20045004 PMC2837789

[B23] Dong E Han Y Sui LN Diabetic retinopathy: VEGF, bFGF and retinal vascular pathology Song Chin Med J 2004 117 247 251 14975211

[B24] Xie XS Liu HC Wang FP Zhang CL Yao CZ Fan JM Ginsenoside Rg_1_modulation on thrombospondin-1 and vascular endothelial growth factor expression in early renal Phytother Res 2010 24 1581 1587 10.1002/ptr.3190 21031612

[B25] Das S Falchi M Bertelli A Maulik N Das DK Attenuation for ischemia/reperfusion injury in rats by the anti-inflammatory action of resveratrol Arzneim Ittelforschung 2006 56 700 706 10.1055/s-0031-1296776 17225566

[B26] Gu B Zhang YD Hu G Antioxidation of metabotropic glutamate receptors ligang on unilateral substantia nigral 6-hydroxydopamine-lesioned rat Chin Pharm Bull 2003 19 41 44

[B27] Daya S Walker R Anoopkumar DS Cyanide Induced free radical production and lipid peroxidation in rat brain homogenate is reduced by aspirin Metab Brain Dis 2000 15 203 210 11206589 10.1007/BF02674529

[B28] Zheng SY Sun J Zhao X Xu JG Protective effect of Shen-Fu on myocardial ischemia-reperfusion injury in rats Am J Chin Med 2004 32 209 220 10.1142/S0192415X04001874 15315259

