# Supplementary material for: Effective components screening and anti-myocardial infarction mechanism study of the Chinese medicine NSLF6 based on "system to system" mode
Source: J Transl Med. 2012 Feb 8;10:26. doi: 10.1186/1479-5876-10-26 (PMC3305499; doi:10.1186/1479-5876-10-26)
Supplement: Additional file 1 — Supplementary materials. [file 1479-5876-10-26-S1.DOC]

Supplementary Materials for

**Effective components screening and mechanism study of the Chinese medicine NSLF6 based on “system to system” mode**

Qiong-Lin Liang,^1^ Xiao-Ping Liang,^1,2^ Yi-Ming Wang,^1^ Yuan-Yuan Xie,^1^  Rong-Li Zhang,^1^ Xi Chen,^1^ Rong Gao,^1^  Yi-Jun Chen,^1, 2^ Jun Wu,^3^ Qing-Bo Xu,^4^ Qing-Zhong Xiao,^5^ Xue Li,^1^ Shu-Feng Lv,^1, 2^ Xue-Mei Fan,^1^ Hong-Yang Zhang,^1, 2^ Qing-Li Zhang,^1,2^ Guo-An Luo ^1, 2^*

*To whom corresponding should be addressed. E-mail: luoga@tsinghua.edu.cn

**SUPPLEMENTARY METERIALS**

**Supplementary methods**

**Chemome Profile and Serum Pharmacochemistry Study of NSLF6**

The shuanglong formula (SLF) was a combination of *panax ginseng* (PG) and *salvia miltiorrhiza* (SM) (at a ratio of 7:3). Dried and pulverized materials of PG (500 g), SM (500 g), and SLF (500 g) were ground and then refluxed with 4000 ml of water for 60 min, twice respectively. After cooling, the extracting solutions were filtered through a glass filter covered with filter paper. The solutions were condensed under decompression to roughly 150 ml and finally were freeze-dried. The extract of ginsenosides (Lot 20090220, EG) and extract of total salvianolic acids (Lot 20100701, ESA) were semi-works products, which were prepared by Guangzhou Xiangxue Pharmaceutical Co., Ltd. The EG and ESA were combined at a ratio of which correspongding to the material medica 7:3). Each of the TCM extracts (SLF and NSLF6) was dissolved in a certain amount of water to a concentration equal to 0.5 g of crude botanicals per milliliter of test solution.

Male Sprague-Dawley rats were randomly divided into five groups each of six rats as follows: SLF group, NSLF6 group, PG group, SM group and normal group, with oral gavage after fasted in 12 hours with the test solutions of PG, SM, SLF, and NSLF6 at a dose of 3.75 g/kg·w·d (equal to 15 ml/ kg·w·d) and the same volume of water, respectively.

Blood samples were collected from the hepatic portal vein of rats in 45 minutes after administered, under 1% pentobarbital sodium anesthesia into microfuge tubes containing heparin as an anticoagulant, and immediately centrifuged at 3000 rpm for 10 min at room temperature. 2 ml methanol was added into 400 μl supernatant and the mixture was vortex-mixed for 2 min followed by centrifugation at 6,000 rpm for 10 min at 4 °C. The clear supernatant was transferred and injected onto analytical column.

Chromatographic separation was performed on an Acquity UPLC BEH C18 column (2.1 × 100 mm, 1.7 μm, Waters Corp., Milford, USA) using an ACQUITYTM UPLC system (Waters), equipped with a binary solvent delivery system, an autosampler, and a PDA detector. The column was maintained at 50 °C and eluted at a flow rate of 0.4 ml/min, using a mobile phase of (A) 0.1% (by volume) formic acid in water and (B) acetonitrile. The gradient program was optimized as follows: 0-4 min, 5% B to 5%B; 4-9 min, 5% B to 50% B; 9-19 min, 50% B to 90% B; 19-20 min, 90% B to 90% B; 20-22 min, 90% B to 5% B; 22-28 min, equilibration with 5% B. The column eluent was directly inducted into the mass spectrometer without split.

Mass spectrometry was performed on a Waters LCT Premier orthogonal accelerated time of flight mass spectrometer (Waters, Millford, MA) with an electrospray ionization source (ESI) operating in negative ion mode (W mode of operation). The capillary voltage and the cone voltage were set at 2200 V and 35 V, respectively. Nitrogen was used as the drying gas. The desolvation gas rate was set to 700 l/h at a temperature of 350 °C, and the cone gas rate was set at 40 l/h and the source temperature was set at 120 °C. The scan time and inter-scan delay were set to 0.2 s and 0.02 s, respectively. Data was collected in centroid mode from m/z 100 to m/z 1500 with a LockSpray frequency of 10 s, and data averaging over 10 scans. The total ion chromatograms of test solutions prepared from selected TCM materials were shown in Table S1.

As a result, seventy three compounds of SLF were identified on the basis of retention time, m/z and available conference standards, among which twenty five compounds were from SM and forty eight compounds from PG. Study of Serum Pharmacochemistry showed that 27 components represented two types of components (ginsenosides and salvianolic acid) in SLF were able to determine in the plasma of rats after administration, which means that these compounds were the material basis for efficacy.

**Quantitive Fingerprint Study of NSLF6**

Chromatographic separation of quantified fingerprint of ginsenosides was performed on an Agilent TC-C_18_ column (4.6 mm × 250 mm, 5 μm Agilent Corp., Milford, USA) using an Agilent1200series HPLC system (Agilent), equipped with a binary solvent delivery system, an autosampler, and a VWD detector. The column was maintained at room temperature and eluted at a flow rate of 1.0 ml/min, using a mobile phase of (A) 2 mmol potassium dihydrogen phosphate in water and (B) acetonitrile. The gradient program was optimized as follows: 0-32 min, 21% B to 23%B; 32-70 min, 23% B to 38% B; 70-80 min, 38% B. The detective wavelength was set at 205 nm.

Chromatographic separation of quantified fingerprint of salvianolic acid was performed on an Alltimal C_18_ column (4.6 mm × 250 mm, 5 μm) using an Shimadzu 20AT HPLC system (Shimadzu), equipped with a binary solvent delivery system, an autosampler, and a PDA detector. The column was maintained at room temperature and eluted at a flow rate of 1.0 ml/min, using a mobile phase of (A) 0.4% (by volume) formic acid in water and (B) acetonitrile. The gradient program was optimized as follows: 0-6 min, 15% B to 15%B; 6-40 min, 15% B to 41% B. The detective wavelength was set at 280 nm.

Supplementary Figure 1

**Biological response system (living system)**

**External intervention system (TCM)**

Supplementary Figure 1. “System to system” mode.

Supplementary Figure 2


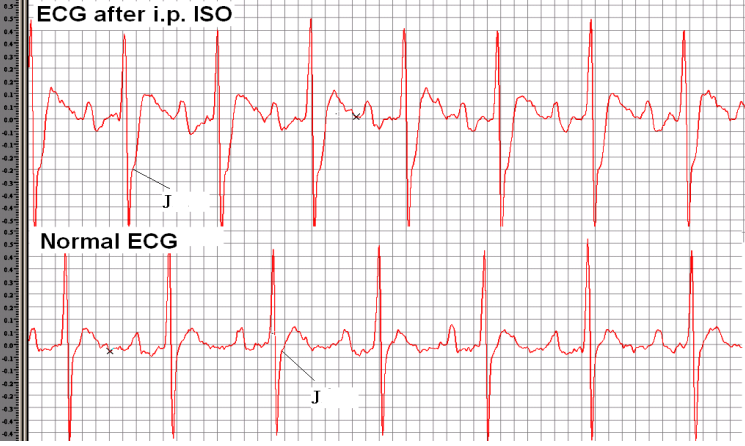


Supplementary Figure 2. Changes of electrocardiogram of rats after isoproterenol injection. Details of the experimental procedures are given in Materials and Methods.

Supplementary Figure 3

**
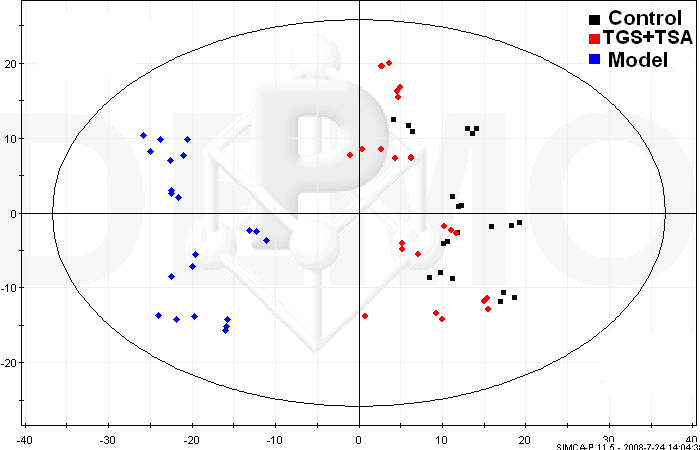
**

Supplementary Figure 3. The PCA score plot of rat serum from control, model and TGS+TSA group. Details of the experimental procedures are given in Materials and Methods.

Supplementary Figure 4

**B**

Supplementary Figure 4. The percentage of definite components in SLF and NSLF6. (A) SLF. (B) NSLF6. Details of the experimental procedures are given in Supplementary methods

Supplementary Figure 5


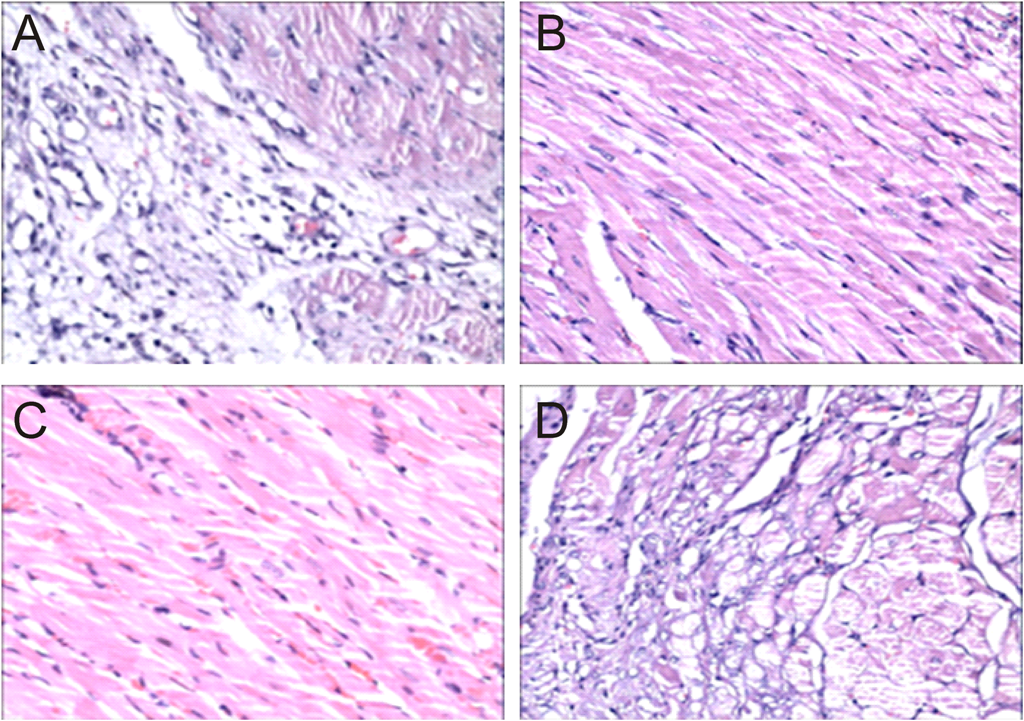


Supplementary Figure 5. Effect of NSLF6 on coronary artery ligation induced myocardial injury. Hematoxylin and eosin staining was used to visualize the formalin-fixed sections of cardiac muscle tissue of rats (magnification 100 diameters). (A) Model group. (B) Sham surgery group. (C) NSLF6 group. (D) Positive control drug group: Diltiazem hydrochloride. Details of the experimental procedures are given in Materials and Methods.

Supplementary Figure 6


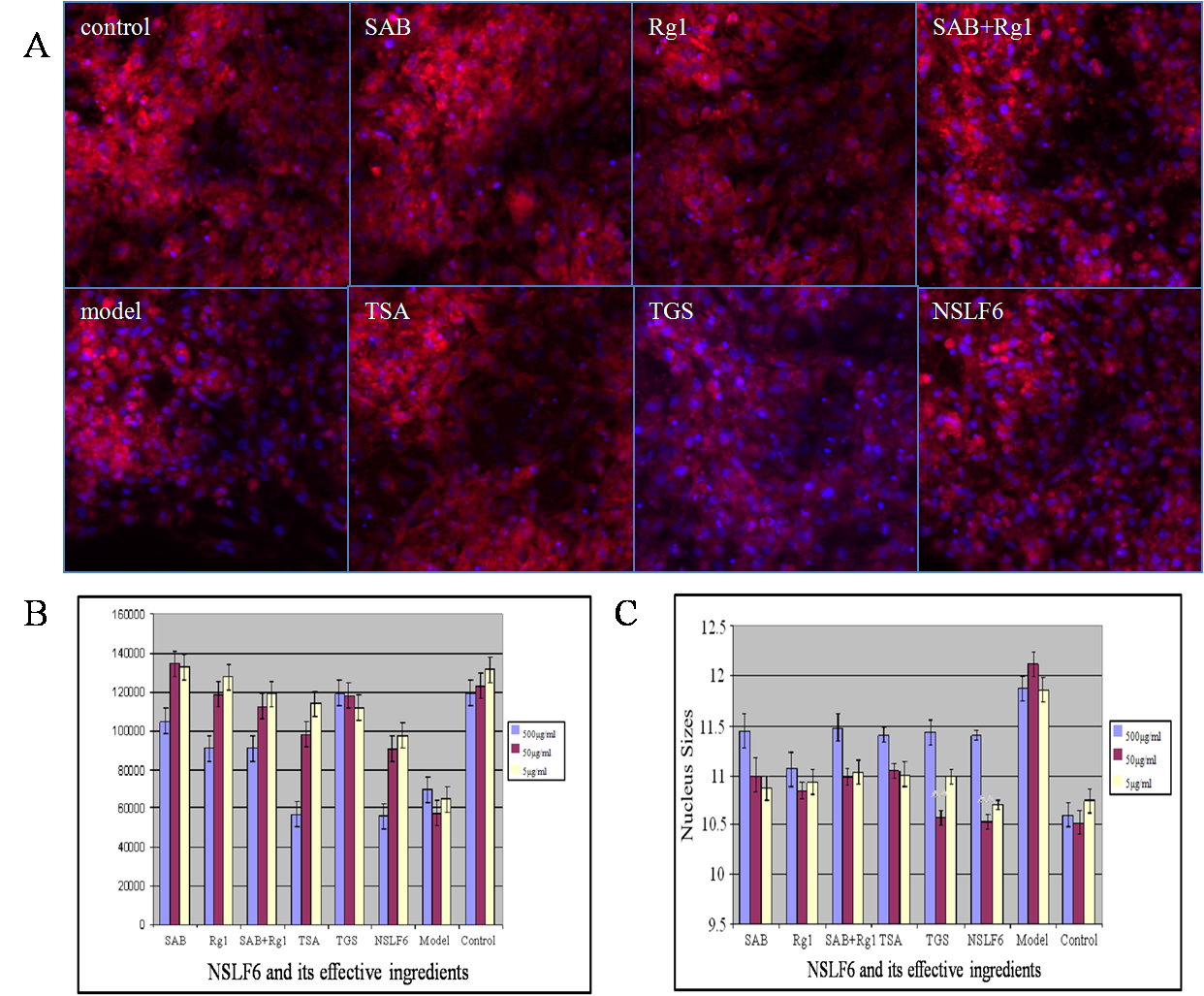


Supplementary Figure 6. The effect of NSLF6 and its effective ingredients to the mitochondrial membrane potential and Nuclear integrity of cardiomyocytes damaged by H_2_O_2_. (A) Fluorescence expressions of the mitochondrial membrane potential and Nuclear integrity of the damaged cardiomyocytes stimulated by NSLF6 and its effective ingredients. Red fluorescence shows the mitochondrial membrane potential, blue fluorescence shows nucleus stained by DAPI. (B) Comparison of NSLF6 and its effective ingredients to the mitochondrial membrane potential of cardiomyocytes damaged by H_2_O_2_. (C) Comparison of NSLF6 and its effective ingredients to the Nuclear integrity of cardiomyocytes damaged by H_2_O_2_ membrane potential of cardiomyocytes damaged by H_2_O_2_. Details of the experimental procedures are given in Materials and Methods.

Supplementary Figure 7


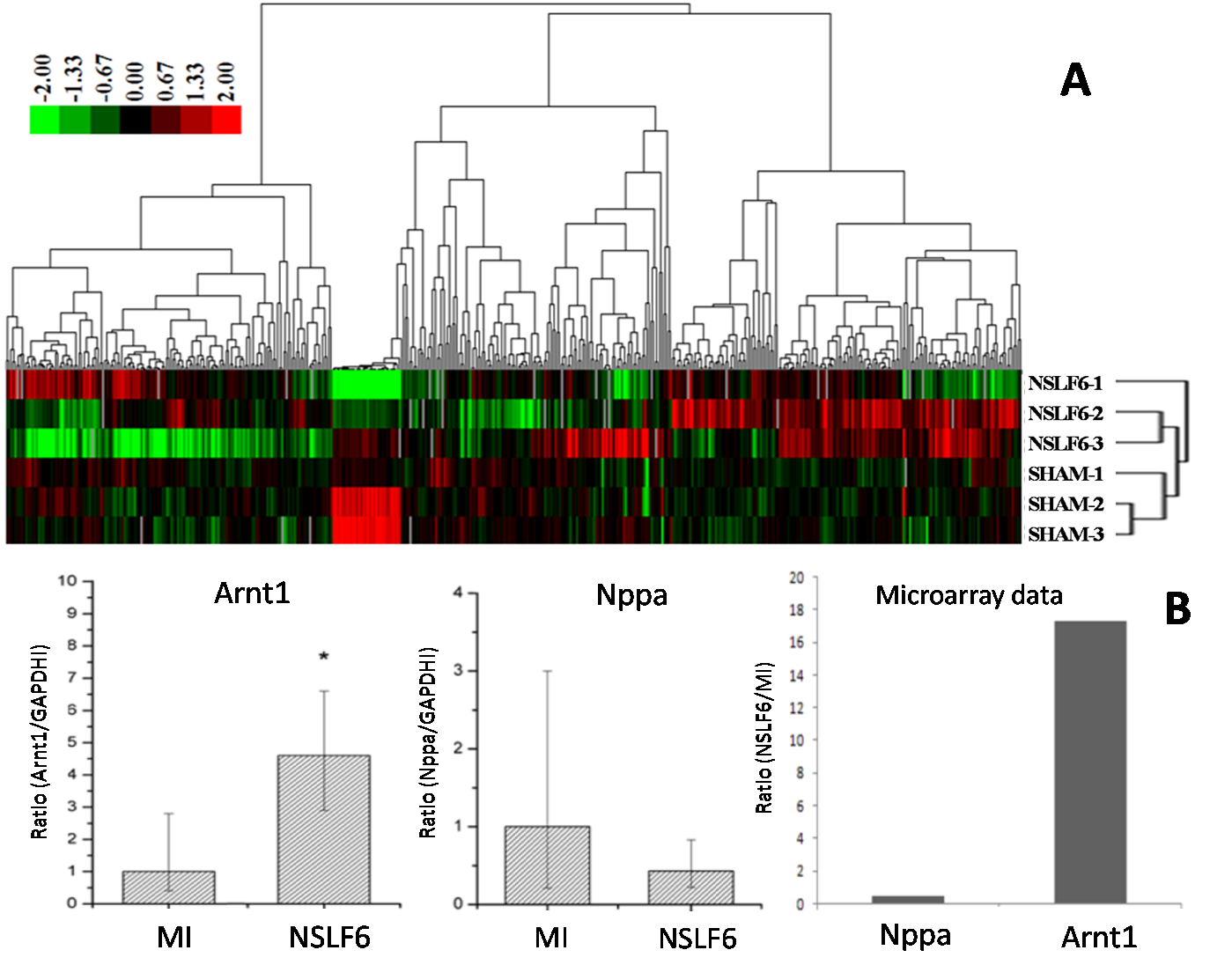


Supplementary Figure 7. The differentially expressed genes cluster analysis and PCR verification. (A) The result of cluster analysis, the red means increased gene expression and the green means reduced gene expression. (B) The expression of Amrt1and Nppa was verified by RT-PCR. The PCR Determination is consistent with microarray analysis. * *P* < 0.05 versus MI. Details of the experimental procedures are given in Materials and Methods.

Supplementary Figure 8


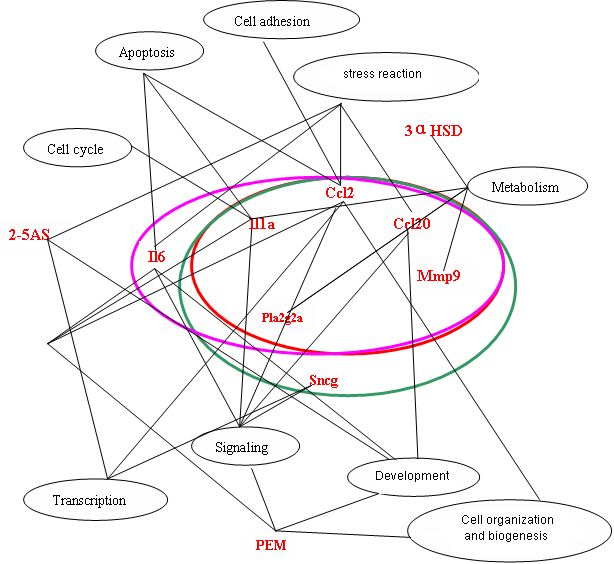


Supplementary Figure 8. The functional relationship of the 10 differentially expressed genes related to the differentiation from BMMSCs into cardiomyocyte-like cells induced by NSLF6. Details of the experimental procedures are given in Materials and Methods.

Supplementary Figure 9


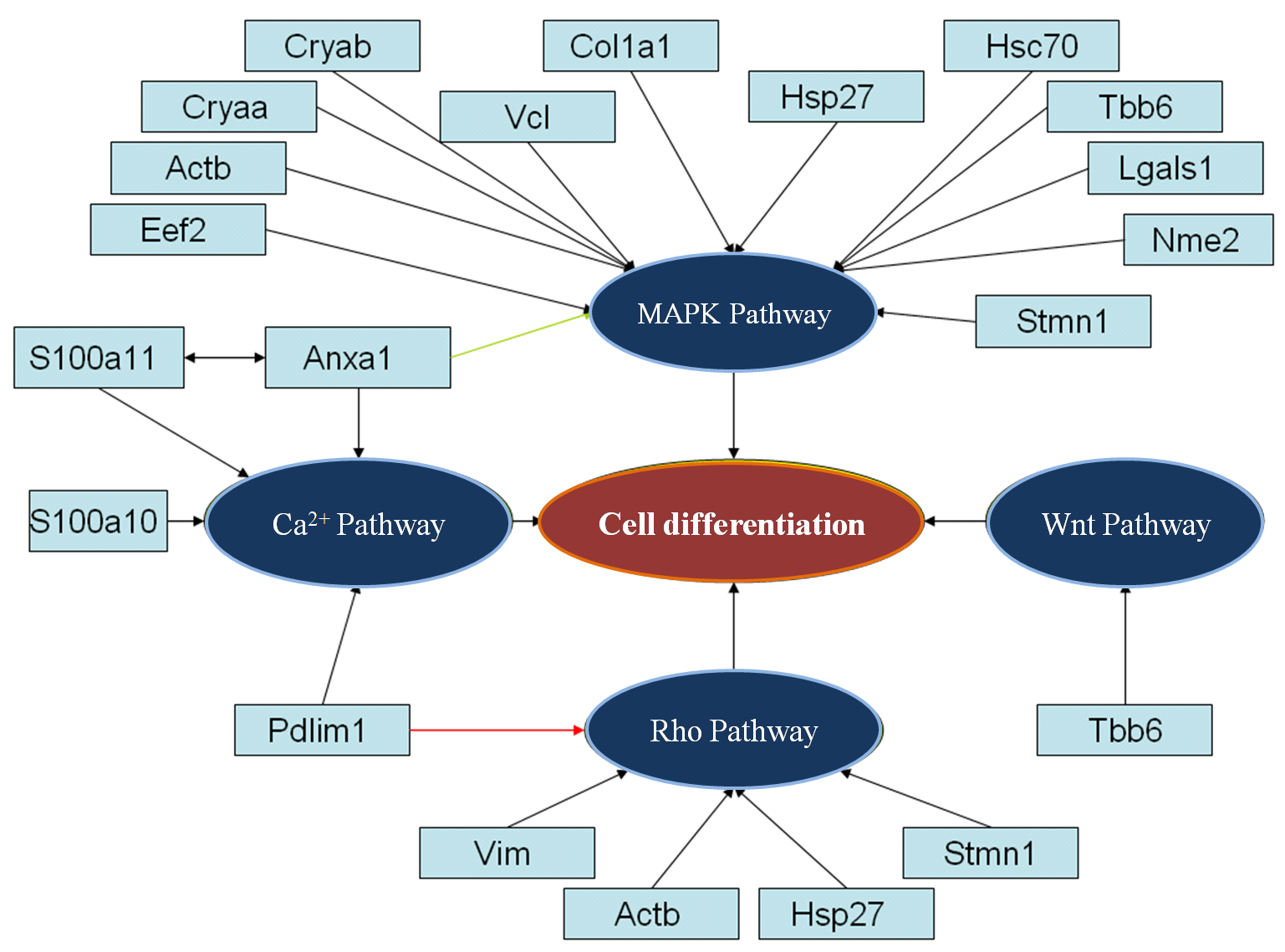


Supplementary Figure 9. The relational signaling pathway network. Details of the experimental procedures are given in Materials and Methods.

Supplementary Table 1

TableS 1 Sequence information of the primers

| Gene | Forward Primer | Reverse Primer |  |
| --- | --- | --- | --- |
| Anrt1 | GTCGAATGATTGCCGAGGAA | GCCAGCGGAAGGAATGTCT |  |
| Nppa | ACCAGAGAGTGAGCCGAGACA | AGCCAAAAGGCCAGGAAGAG |  |
| GAPDH | GCATCCTCGGCTACAGTGAG | TCCACCACCATGTTGCTGTA |  |

Details of the experimental procedures are given in Supplementary Materials.

Supplementary Table 2

Supplementary Table 2. Effects of SLF on ΔJ of electrocardiogram in MI rats induced by isoproterenol at different time points (x ± s, *n* = 9).

| Group | Dose (g/kg·d) | 5 min | 10 min | 15 min | 20 min |
| --- | --- | --- | --- | --- | --- |
| Model | - | -0.167 ± 0.091 | -0.176 ± 0.101 | -0.179 ± 0.098 | -0.194 ± 0.086 |
| Control | - | 0.009 ± 0.021*** | 0.004 ± 0.025*** | 0.003 ± 0.029*** | 0.003 ± 0.031*** |
| Positive control | 2 | -0.133 ± 0.158 | -0.123 ± 0.147 | -0.118 ± 0.149 | -0.108 ± 0.136 |
| PG:SM (6:4) | 5 | -0.095 ± 0.093 | -0.077 ± 0.099* | -0.089 ± 0.099* | -0.070 ± 0.093*** |
| PG:SM (7:3) | 5 | -0.060 ± 0.070** | -0.061 ± 0.062** | -0.064 ± 0.057** | -0.054 ± 0.061*** |
| PG:SM (8:2) | 5 | -0.081 ± 0.101 | -0.054 ± 0.138** | -0.052 ± 0.130** | -0.051 ± 0.142*** |
| SM | 5 | -0.155 ± 0.103 | -0.151 ± 0.126 | -0.140 ± 0.114 | -0.118 ± 0.112 |
| PG | 5 | -0.093 ± 0.086 | -0.100 ± 0.076 | -0.108 ± 0.110 | -0.087 ± 0.080 |

* *P* < 0.05, ** *P* < 0.01, *** *P* < 0.001 vs model group. Positive control drug: Diltiazem hydrochloride. Details of the experimental procedures are given in Materials and Methods.

Supplementary Table 3

Supplementary Table 3. Effects of SLF on the activities of LDH, CK, SOD, and MDA in serum of MI rats induced by isoproterenol (x ± s, *n* = 9).

| Group | Dose (g/kg·d ) | LDH  (µmol·mL^-1^) | CK  (µmol·mL^-1^) | SOD  (µmol·mL^-1^) | MDA  (µmol·L^-1^) |
| --- | --- | --- | --- | --- | --- |
| Model | - | 0.27 ± 0.12 | 4.24 ± 1.79 | 44.55 ± 6.09 | 5.51 ± 1.30 |
| Control | - | 0.12 ± 0.06** | 2.12 ± 0.72** | 50.90 ± 3.96* | 2.18 ± 0.80*** |
| Positive control | 2 | 0.22 ± 0.08 | 3.01 ± 0.77 | 47.34 ± 5.87 | 4.10 ± 1.84 |
| PG:SM (6:4) | 5 | 0.13 ± 0.07* | 2.26 ± 1.07* | 49.65 ± 5.00* | 3.85 ± 1.38** |
| PG:SM (7:3) | 5 | 0.15 ± 0.07* | 2.64 ± 1.08* | 50.41 ± 4.79* | 3.91 ± 1.73* |
| PG:SM (8:2) | 5 | 0.15 ± 0.08* | 2.72 ± 1.15* | 49.65 ± 3.93* | 3.79 ± 1.59* |
| SM | 5 | 0.18 ± 0.09 | 3.81 ± 1.43 | 47.55 ± 6.36 | 4.31 ± 1.63 |
| PG | 5 | 0.15 ± 0.10* | 2.89 ± 1.40 | 47.05 ± 7.61 | 4.30 ± 2.00 |

* *P* < 0.05, ** *P* < 0.01, *** *P* < 0.001 vs model group. Positive control drug: Diltiazem hydrochloride. Details of the experimental procedures are given in Materials and Methods.

Supplementary Table 4

Supplementary Table 4. Effects of SLF on cardiac infarction area in MI rats induced by isoproterenol (x ± s, *n* = 9).

| Group | Dose (g/kg·d) | Cardiac infarction area % |
| --- | --- | --- |
| Model | - | 9.94 ± 4.19 |
| Control | - | 1.01 ± 0.87*** |
| Positive control | 2 | 7.72 ± 2.36 |
| PG:SM (6:4) | 5 | 6.91 ± 2.03* |
| PG:SM (7:3) | 5 | 6.87 ± 2.93* |
| PG:SM (8:2) | 5 | 6.70 ± 2.12* |
| SM | 5 | 7.65 ± 3.11 |
| PG | 5 | 8.10 ± 3.86 |

* *P* < 0.05, *** *P* < 0.001 vs model group. Positive control drug: Diltiazem hydrochloride. Details of the experimental procedures are given in Materials and Methods.

Supplementary Table 5

Supplementary Table 5. List of global chemome for SLF

| No. | R.T.  (min) | Selected  ion | m/z | Elemental  composition | In vivo | In vitro |
| --- | --- | --- | --- | --- | --- | --- |
| 1 | 0.54 | [M-H] | 197.0272 | C_9_H_10_O_5_ | 3-(3,4-dihydroxyhenyl) lactic acid | propanoid acid |
| 2 | 0.64 | [M-H] | 313.0923 | C_17_H_14_O_6_ | salvianolic acid F |  |
| 3 | 1.26 | [M-H] | 137.0224 | C_7_H_6_O_3_ | protocatechuic aldehyde | protocatechualdehyde |
| 4 | 1.55 | [M-H] | 383.0956 | C_21_H_20_O_7_ | salvianolic acid K |  |
| 5 | 1.62 | [M+COOH] | 315.1078 | C_27_H_22_O_12_ | salvianolic acid H | isoimperatrin |
| 6 | 1.83 | [M-H] | 417.1034 | C_20_H_18_O_10_ | salvianolic acid I |  |
| 7 | 1.87 | [M-H] | 371.0975 | C_19_H_16_O_8_ | salvianolic acid D |  |
| 8 | 2.31 | [M-H] | 537.1421 | C_27_H_22_O_12_ | salvianolic acid M |  |
| 9 | 2.70 | [M-H] | 555.1463 | C_27_H_24_O_13_ | salvianolic acid G |  |
| 10 | 2.80 | [M-H] | 359.1027 | C_18_H_16_O_8_ | salvianolic acid L |  |
| 11 | 2.80 | [M-H] | 717.2053 | C_36_H_30_O_16_ | salvianolic acid F |  |
| 12 | 2.91 | [M-H] | 537.1498 | C_27_H_22_O_12_ | rosmarinic acid | salvianolic acid I |
| 13 | 3.30 | [M-H] | 717.2090 | C_36_H_30_O_16_ | salvianolic acid J |  |
| 14 | 3.43 | [M-H] | 537.1498 | C_27_H_22_O_12_ | ginsenoside Rs2 |  |
| 15 | 3.69 | [M-H] | 445.0963 | C_21_H_18_O_11_ | lithospermic acid |  |
| 16 | 3.71 | [M-H] | 491.1032 | C_26_H_22_O_10_ | salvianolic acid B | salvianolic acid C |
| 17 | 3.80 | [M-H] | 717.2072 | C_36_H_30_O_16_ | ginsenoside F4 |  |
| 18 | 3.87 | [M-H] | 455.3541 | C_30_H_48_O_3_ | 20-gluco-ginsenoside Rf |  |
| 19 | 3.87 | [M+COOH] | 831.4751 | C_41_H_70_O_14_ | salvianolic acid E |  |
| 20 | 4.06 | [M-H] | 419.0966 | C_19_H_18_O_8_ | Notoginsenoside-R1 |  |
| 21 | 4.12 | [M+COOH] | 731.2222 | C_31_H_50_O_3_ | 9’-methyl lithospermate B |  |
| 22 | 4.17 | [M+COOH] | 1007.6335 | C_48_H_82_O_19_ | methyl rosmarinate |  |
| 23 | 4.41 | [M+COOH] | 977.5273 | C_47_H_80_O_18_ | 9’ methyl salvianolic acid B | notoginsenoside-R_1_ |
| 24 | 4.51 | [M+COOH] | 519.3562 | C_30_H_50_O_4_ | ginsenoside Re | protopanaxatriol |
| 25 | 4.59 | [M+COOH] | 537.1498 | C_27_H_22_0_12_ | lithospermic acid/isomer |  |
| 26 | 4.77 | [M-H] | 493.1253 | C_26_H_22_O_10_ | salvianolic acid A | salvianolic acid A |
| 27 | 4.83 | [M+COOH] | 991.5528 | C_48_H_82_O_18_ | ginsenoside Re |  |
| 28 | 4.89 | [M+COOH] | 845.5647 | C_42_H_72_O_14_ | ginsenoside Rf | ginsenoside Rf |
| 29 | 4.90 | [M+COOH] | 459.3927 | C_29_H_50_O | β- sitosterol |  |
| 30 | 5.16 | [M-H] | 885.5041 | C_45_H_74_O_17_ | ginsenoside malonyl- Rg1 | ginsenoside malonyl- Rg1 |
| 31 | 5.25 | [M-H] | 491.0979 | C_19_H_16_O_8_ | isosalvianolic acid C | isosalvianolic acid C |
| 32 | 5.44 | [M-H] | 1031.5356 | C_51_H_84_O_21_ | N.I. |  |
| 33 | 5.55 | [M-H] | 885.5643 | C_45_H_74_O_17_ | malonyl- Rg1 isomer |  |
| 34 | 6.15 | [M+COOH] | 815.4805 | C_41_H_70_O_13_ | ginsenoside F3 |  |
| 35 | 6.38 | [M-H] | 745.2044 | C_27_H_24_O_13_ | dimethyl salvianolate B |  |
| 36 | 6.67 |  | 1033.6500 |  | N.I. |  |
| 37 | 7.09 | [M+COOH] | 845.5662 | C_42_H_72_O_14_ | ginsenoside Rg1 | ginsenoside Rg1 |
| 38 | 7.41 | [M-H] | 377.1961 | C_21_H_29_O_6_ | N.I. | propanoid acid-gluA |
| 39 | 7.33 | [M+COOH] | 1285.6573 | C_59_H_100_O_27_ | Notoginsenoside-R4 |  |
| 40 | 7.47 | [M+COOH] | 815.5520 | C_41_H_70_O_13_ | Notoginsenoside-R2 |  |
| 41 | 7.56 | [M-H] | 329.2471 | C_20_H_26_O_4_ | salviol | salviol |
| 42 | 7.88 | [M+COOH] | 829.5691 | C_42_H_72_O_13_ | ginsenoside Rg2 |  |
| 43 | 7.90 | [M+COOH] | 815.4859 | C_41_H_70_O_13_ | ginsenoside F5 |  |
| 49 | 8.18 | [M+COOH] | 1075.6365 | C_55_H_92_O_23_ | ginsenoside Rs2 |  |
| 50 | 8.19 | [M+COOH] | 931.6228 | C_54_H_94_O_9_ | N.I. | Compound K oleate |
| 51 | 8.27 | [M+COOH] | 1001.5022 | C_49_H_80_O_18_ | ginsenoside R_0_ |  |
| 52 | 8.28 | [M+COOH] | 683.4992 | C_36_H_62_O_9_ | ginsenoside F1 | ginsenoside F1 |
| 53 | 8.42 | [M+COOH] | 1255.6363 | C_58_H_98_0_26_ | ginsenoside Ra2 |  |
| 54 | 8.47 | [M+COOH] | 1123.5933 | C_53_H_90_O_22_ | ginsenoside Rb2 | ginsenoside Rb2 |
| 55 | 8.50 | [M-H] | 1165.5982 | C_56_H_92_O_25_ | ginsenoside malonyl- -Rb2 | ginsenoside malonyl-Rb2 |
| 56 | 8.77 | [M+COOH] | 1123.5909 | C_53_H_90_O_22_ | ginsenoside malonyl- Rb3 |  |
| 57 | 8.80 | [M+COOH] | 1123.5868 | C_53_H_90_O_22_ | ginsenoside Rc | ginsenoside Rc |
| 58 | 8.88 | [M-H] | 1165.5994 | C_56_H_92_O_25_ | ginsenoside malonyl- -Rc | ginsenoside malonyl-Rc |
| 59 | 8.96 | [M-H] | 339.0806 | C_18_H_12_O_7_ | salvianolic acid G | salvianolic acid |
| 60 | 9.16 | [M+COOH] | 1195.6102 | C_56_H_94_O_24_ | panas quinquefolium R1 |  |
| 61 | 9.19 | [M-H] | 1093.5857 | C_53_H_90_O_23_ | N.I. |  |
| 62 | 9.50 | [M+COOH] | 1165.7053 | C_55_H_92_O_23_ | ginsenoside Rs1 |  |
| 63 | 9.56 | [M+COOH] | 991.5594 | C_48_H_82_O_18_ | ginsenoside Rd | ginsenoside Rd |
| 64 | 9.82 | [M+COOH] | 503.2664 | C_30_H_50_O_3_ | protopanoxadiol | protopanoxadiol |
| 65 | 9.84 | [M+COOH] | 521.3710 | C_30_H_52_O_4_ | N.I. | protopanaxatriol |
| 66 | 10.10 | [M+COOH] | 991.5609 | C_48_H_82_O_18_ | ginsenoside Rd isomer |  |
| 67 | 10.56 | [M-H] | 961.5531 | C_48_H_82_O_19_ | 220-gluco-ginsenoside Rf isomer |  |
| 68 | 10.72 |  | 1033.5769 |  | N.I. |  |
| 69 | 10.91 |  | 961.6228 |  | N.I. |  |
| 70 | 11.16 | [M+COOH] | 811.5568 | C_42_H_70_O_12_ | ginsenoside Rg4 |  |
| 71 | 11.43 | [M+COOH] | 811.5567 | C_42_H_70_O_12_ | ginsenoside Rg5 |  |
| 72 | 11.81 |  | 665.4831 |  | N.I. |  |
| 73 | 11.96 | [M+COOH] | 829.4880 | C_42_H_72_O_13_ | ginsenoside Rg2 isomer |  |
| 74 | 12.62 | [M+COOH] | 505.3546 | C_30_H_52_O_3_ | protopanoxadiol | protopanoxadiol |
| 75 | 12.70 | [M+COOH] | 829.5677 | C_42_H_72_O_13_ | ginsenoside F2 |  |
| 76 | 12.92 | [M+COOH] | 829.5697 | C_42_H_72_O_13_ | ginsenoside Rg3 |  |
| 77 | 13.32 | [M+COOH] | 799.4894 | C_41_H_70_O_12_ | ginsenoside MC |  |
| 78 | 13.91 |  | 595.2895 |  | N.I. |  |
| 79 | 14.43 |  | 1191.5946 |  | N.I. |  |
| 80 | 14.69 |  | 723.4442 |  | N.I. |  |
| 81 | 15.03 | [M+COOH] | 667.4415 | C_36_H_62_O_8_ | ginsenoside Rh2 | ginsenoside Rh2 |
| 82 | 15.11 | [M+COOH] | 851.4934 | C_51_H_98_O_6_ | tripalmitin |  |
| 83 | 15.21 | [M+COOH] | 811.4869 | C_42_H_70_O_12_ | ginsenoside Rg6 |  |
| 84 | 15.43 | [M+COOH] | 649.3914 | C_36_H_60_O_7_ | ginsenoside Rh3 |  |
| 85 | 15.48 | [M+COOH] | 811.4849 | C_42_H_70_O_12_ | ginsenoside Rk1 |  |
| 86 | 15.58 | [M+COOH] | 667.4438 | C_36_H_62_O_8_ | Compound K | Compound K |
| 87 | 15.97 |  | 433.2725 |  | N.I. |  |
| 88 | 16.33 | [M+COOH] | 625.2377 | C_28_H_36_O_13_ | eleutheroside B |  |
| 89 | 17.08 | [M-H] | 455.2816 | C_30_H_48_O_3_ | oleanolic acid |  |
| 90 | 17.46 | [M+COOH] | 461.26677 | C_27_H_44_O_3_ | tigogenin |  |
| 91 | 18.22 | [M+COOH] | 649.4343 | C_36_H_60_O_7_ | ginsenoside Rk2 |  |

Details of the experimental procedures are given in Materials and Methods.

Supplementary Table 6

Supplementary Table 6. Effects of SLF extracts on 2 min ΔJ of ECG in MI rats induced by isoproterenol (x ± s, *n* = 10)

| No. | Group | ΔJ (mv) | T test |
| --- | --- | --- | --- |
| 1 | Model group | -0.1347 |  |
| 2 | Control group | 0.0090 | *P* < 0.001 |
| 3 | TGS + TSA | -0.0421 | *P* < 0.01 |
| 4 | TGS + TSA + RPG | -0.0404 | *P* < 0.01 |
| 5 | TGS + TSA + RSM | -0.0625 | *P* < 0.05 |
| 6 | TSA + RPG + RSM | -0.0986 | *P* > 0.05 |
| 7 | TGS + RPG + RSM | -0.1480 | *P* > 0.05 |
| 8 | RPG + RSM | -0.1613 | *P* > 0.05 |
| 9 | TGS + TSA + RPG + RSM | -0.0194 | *P* < 0.01 |
| 10 | Positive control | -0.0881 | *P* > 0.05 |

TGS: total ginsenosides, RPG: the remainder of PG (mainly containing panaxan, TGS removed), TSA: total salvianolic acids, RSM: the remainder of SM (mainly containing diterpenoid quinones, TSA removed). Positive control drug: Diltiazem. *P* < 0.05, *P* < 0.01, *P* < 0.001 vs model group. Details of the experimental procedures are given in Materials and Methods.

Supplementary Table 7

Supplementary Table 7 Contents of major constituents in SLF and NSLF6.

| Components | SLF (%) | NSLF6 (%) |
| --- | --- | --- |
| Ginsenoside Rg_1_ | 0.29 | 3.95 |
| Ginsenoside Re | 0.18 | 3.72 |
| Ginsenoside Ro | 0.28 | 7.91 |
| Ginsenoside m-Rb_1_ | 0.07 | 2.33 |
| Ginsenoside m-Rc | 0.09 | 1.14 |
| Ginsenoside m-Rb_2_ | 0.10 | 1.12 |
| Ginsenoside m-Rd | 0.05 | 0.63 |
| Ginsenoside Rf | 0.04 | 1.29 |
| Ginsenoside Rb_1_ | 0.49 | 9.87 |
| Ginsenoside Rc | 0.19 | 5.44 |
| Ginsenoside Rb_2_ | 0.16 | 4.20 |
| Ginsenoside Rd | 0.10 | 2.28 |
| Propanoid acid | 0.15 | 0.47 |
| Protocatechualdehyde | 0.01 | 0.03 |
| Caffeic acid | 0.04 | 0.19 |
| Salvianolic acid J/Isomer | 0.11 | 0.68 |
| Rosmarinic acid | 0.08 | 0.89 |
| Lithospermic acid | 0.14 | 2.70 |
| Salvianolic acid B | 1.64 | 24.7 |
| Salvianolic acid E | 0.09 | 0.80 |
| Total | 4.30 | 74.4 |

Details of the experimental procedures are given in Supplementary methods

Supplementary Table 8

Supplementary Table 8. Effects of NSLF6 on activities of LDH and CK in serum on MI rats induced by coronary artery ligation (*x* ± *s*).

| Group | Dose (g/kg·d) | Sample | CK (μmol·mL^-1^) | LDH (μmol·mL^-1^) |
| --- | --- | --- | --- | --- |
| Model | - | 9 | 2.15 ± 0.43^#^ | 15.60 ± 2.75^#^ |
| Control | - | 10 | 0.81 ± 0.53 | 8.28 ± 3.20 |
| Sham surgery | - | 10 | 1.36 ± 0.35**^#^ | 8.82 ± 4.07** |
| NSLF6 | 5 | 11 | 0.86 ± 0.32** | 9.02 ± 2.20** |
| Positive control | 0.05 | 7 | 1.37 ± 0.39** | 12.43 ± 1.69*^#^ |

Positive control drug: Diltiazem. **P* < 0.05，** *P* < 0.01 vs model group; ^#^*P* < 0.05 vs control. Details of the experimental procedures are given in Materials and Methods.

Supplementary Table 9

Supplementary Table 9. Effects of NSLF6 on cardiac infarction area in MI rats induced by coronary artery ligation (x ± s).

| Group | Dose (g/kg·d) | Sample | Cardiac infarction area (%) |
| --- | --- | --- | --- |
| Model | - | 10 | 22.04 ± 3.84 |
| NSLF6 | 5 | 10 | 9.16 ± 1.37** ^#^ |
| Positive control | 0.05 | 10 | 16.05 ± 3.71 |

Positive control drug: Diltiazem hydrochloride. Details of the experimental procedures are given in Materials and Methods.

Supplementary Table 10

Supplementary Table 10. Effects of NSLF6 on the viability of myocardiocyte damaged by H/R.

|  | MI | TSG | TSA | NSLF6 | DH |
| --- | --- | --- | --- | --- | --- |
| Absorbance for MTT(AU) | 0.036 ± 0.008 | 0.25 ± 0.05 | 0.11 ± 0.04 | 0.31 ± 0.06 | 0.073 ± 0.01 |
| Viability (% control) | 8.26 | 57.57* | 24.77* | 70.41* | 16.74* |

* *P* < 0.01vs MI model group. Details of the experimental procedures are given in Materials and Methods.

Supplementary Table 11

Supplementary Table 11. Identification of significantly differential endogenous metabolites in the urine of coronary artery ligation induced MI rats treated with NSLF6.

| ID | R.T  (min) | Selected ion | Mass(m/z) | Elemental composition | Identification by MS | Day 0 | Day 1 | Day 7 | Day 14 |
| --- | --- | --- | --- | --- | --- | --- | --- | --- | --- |
| 1 | 9.08 | [M-H]^-^ | 595.2056 | C_36_H_40_N_2_O_6_ | urobilinogen | 333.26 | 49.00↓ | 160.01 | 243.93 |
| 2 | 4.30 | [M-H]^-^ | 192.0601 | C_10_H_11_NO_3_ | 2-methylhippuric acid | 145.98 | 82.20↓ | 105.23 | 123.35 |
| 3 | 8.77 | [M-H]^-^ | 201.0161 | C_7_H_7_O_5_P | benzoylphosphate | 145.34 | 31.57↓ | 94.71 | 100.68 |
| 4 | 2.73 | [M-H]^-^ | 211.9975 | C_8_H_7_NO_4_S | L-aspartyl-4-phosphate | 321.96 | 67.04↓ | 193.13 | 329.32 |
| 5 | 3.79 | [M-H]^-^ | 242.9937 | C_9_H_12_N_2_O_6_ | unknown | 13.49 | 80.64↑ | 60.24 | 48.76 |
| 6 | 3.34 | [M-H]^-^ | 245.0096 | C_13_H_14_NO_3_ | N-acetyl-D-tryptophan | 355.00 | 36.98↓ | 267.41 | 260.32 |
| 7 | 0.66 | [M+COOH]- | 254.9791 | C_6_H_10_O_8_ | Glucaric acid | 121.55 | 184.73↑ | 111.77 | 102.43 |
| 8 | 3.21 | [M-H]^-^ | 273.0051 | C_6_H_11_O_10_ P | D-glucuronic acid 1-phosphate | 114.43 | 349.74↑ | 104.20 | 102.71 |
| 9 | 5.98 | [M-H]^-^ | 283.0793 | C_10_H_12_N_4_O_6_ | xanthosine | 235.83 | 45.36↓ | 137.40 | 182.92 |
| 10 | 4.29 | [M+COOH]^-^ | 385.1398 |  | unknown | 255.59 | 176.35↓ | 169.79 | 220.05 |
| 11 | 5.96 | [M-H]^-^ | 567.1706 | C_30_H_48_O_10_ | Deoxycholic acid 3-glucuronide | 239.91 | 51.74↓ | 155.13 | 220.57 |
| 12 | 4.46 | [M-H]^-^ | 648.9041 | C_10_H_17_N_5_O_17_P_4_ | N-ligoceroylsphingosine | 145.93 | 57.72↓ | 76.32 | 103.78 |
| 13 | 9.09 | [M-H]^-^ | 297.0969 | C_19_H_38_O_2_ | Nonadecanoic acid | 298.85 | 59.01↓ | 211.02 | 286.32 |
| 14 | 9.12 | [M+COOH]^-^ | 417.1176 |  | unknown | 38.34 | 90.25↑ | 77.65 | 53.16 |
| 15 | 1.62 | [M+COOH]^-^ | 246.9890 | C_8_H_14_N_2_O_4_ | Sebacic acid | 9.43 | 77.68↑ | 14.16 | 8.97 |
| 16 | 2.68 | [M-H]^-^ | 330.0281 | C_10_H_14_N_5_O_6_P | Deoxyadensine monophosphate | 3.01 | 58.59↑ | 10.64 | 1.16 |
| 17 | 3.21 | [M-H]^-^ | 277.0214 | C_12_H_15_N_4_O_2_S | isovaleryglucuronide | 64.31 | 28.19↓ | 32.51 | 44.63 |
| 18 | 3.21 | [M-H]^-^ | 357.1075 | C_11_H_23_N_2_O_7_PS | 2-phenylaminoadenosine | 193.63 | 41.04↓ | 118.15 | 158.43 |
| 19 | 3.24 | [M-H]^-^ | 275.0216 | C_6_H_13_O_10_P | 6-phosphogluconic acid | 8.81 | 156.90↑ | 17.23 | 35.80 |
| 20 | 4.45 | [M-H]^-^ | 646.9079 | C_42_H_81_NO_3_ | ceramide | 94.32 | 204.52↑ | 175.93 | 124.40 |
| 21 | 4.89 | [M+COOH]^-^ | 338.0870 | C_10_H_13_N_5_O_5_ | guanosine | 89.29 | 33.58↓ | 54.00 | 77.29 |
| 22 | 1.07 | [M-H]^-^ | 227.9934 | C_5_H_12_NO_7_P | 5-phosphoribosylamine | 45.35 | 20.41↓ | 30.31 | 47.05 |
| 23 | 1.87 | [M-H]^-^ | 188.9813 | C_6_H_6_O_7_ | oxalosuccinate | 45.03 | 15.88↓ | 41.40 | 38.15 |
| 24 | 3.21 | [M-H]^-^ | 178.0415 | C_9_H_9_NO_3_ | Hippuric acid | 188.68 | 60.85↓ | 167.21 | 140.67 |
| 25 | 4.91 | [M-H]^-^ | 187.0008 | C_7_H_8_O_4_S | N-acetylglutamine | 84.50 | 20.92↓ | 29.80 | 74.60 |
| 26 | 3.70 | [M-H]^-^ | 336.070 | C_11_H_19_N_3_O_7_S | S-(hydroxymethyl)glutathione | 69.98 | 27.69↓ | 57.63 | 41.43 |

Day 1, 7 and 14 mean the time of treatment by NSLF6. “↑” represents a higher level of metabolites, whereas “↓” represents a lower level of metabolites compared to Day 0 (the normal control group). “day 0” presents that the rat urine sample was obtained before coronary artery ligation, “day 1, day 7 and day 14” respectively present that the rat urine sample was obtained at the 1st day, 7th day and 14th day after coronary artery ligation. Details of the experimental procedures are given in Materials and Methods.

Supplementary Table 12

Supplementary Table 12. Identification of significantly differential endogenous metabolites in the serum of ISO-induced MI rat treated with SLF.

| No. | R. T.  (min) | Selected ion | Mass (m/z) | Elemental composition | model | SLF | Positive control | Identification by MS |
| --- | --- | --- | --- | --- | --- | --- | --- | --- |
| 1 | 6.4 | [M+COOH]^–^ | 564.3301 | C_27_H_51_NO_9_P | 4464.96 (↓) | 6072.78 | 5226.69 | Lyso-PC (C18:2) |
| 2 | 6.6 | [M+COOH]^–^ | 612.3301 | C_31_H_51_NO_9_P | 5409.74 (↓) | 6267.72 | 5227.34 | Lyso-PC (C22:6) |
| 3 | 6.7 | [M+COOH]^–^ | 588.3301 | C_29_H_51_NO_9_P | 14244.8 (↓) | 11226.9 | 12514.7 | Lyso-PC (C20:4) |
| 4 | 7.5 | [M+COOH]^–^ | 540.3301 | C_25_H_51_NO_9_P | 59455.1 (↓) | 46986.5 | 52586.8 | Lyso-PC (C16:0) |
| 5 | 8.0 | [M+COOH]^–^ | 566.3458 | C_27_H_53_NO_9_P | 8658.46 (↓) | 9983.50 | 10279.8 | Lyso-PC (C18:1) |
| 6 | 9.1 | [M+COOH]^–^ | 640.3128 | C_13_H_20_N_5_O_16_P_3_ | 3276.38 (↑) | 2960.55 | 3331.33 | unknown |
| 7 | 9.9 | [M-H]^–^ | 480.3090 | C_23_H_47_NO_7_P | 1128.58 (↓) | 4019.89 | 3923.95 | Lyso-PC (C15:1) |
| 8 | 10.0 | [M+COOH]^–^ | 568.3614 | C_27_H_55_NO_9_P | 68080.1 (↑) | 41194.1 | 53028.9 | Lyso-PC (C18:0) |
| 9 | 11.8 | [M-H]^–^ | 327.2329 | C_22_H_31_O_2_ | 6215.17 (↓) | 6743.73 | 7392.36 | Docosahexaenoic acid |
| 10 | 12.1 | [M-H]^–^ | 303.2329 | C_20_H_31_O_2_ | 5406.36 (↑) | 4245.12 | 4505.60 | Arachidonic acid |
| 11 | 12.4 | [M-H]^–^ | 279.2329 | C_18_H_31_O_2_ | 3433.98 (↓) | 4481.87 | 4743.54 | linoleic acid |
| 12 | 13.8 | [M-H]^–^ | 255.2339 | C_16_H_31_O_2_ | 3294.71 (↑) | 1765.8 | 1848.60 | Palmitic acid |
| 13 | 14.3 | [M-H]^–^ | 281.2486 | C_18_H_33_O_2_ | 2458.71 (↓) | 3055.35 | 3587.81 | Oleic acid |
| 14 | 16.4 | [M-H]^–^ | 283.2637 | C_18_H_35_O_2_ | 4841.94 (↑) | 3570.76 | 3449.11 | Stearic acid |

“↑” represents a higher level of metabolites, whereas “↓” represents a lower level of metabolites compared to the normal control group. Details of the experimental procedures are given in Materials and Methods.

Supplementary Table 13

Supplementary Table 13. Proteins identified to be involved in NSLF6-induced differentiation.

| SSP No. | Protein Name (gene name) | AC for databases | MW(kDa) / pI | Score | Matched/all peptides |
| --- | --- | --- | --- | --- | --- |
| 2614 | Vimentin (Vim) | gi|57480 P31000 | 53.76/5.0 | 319 | 41/68 |
| 2713 | Prolyl 4-hydroxylase, beta polypeptide (P4hb) | gi|6981324 P04785 | 57.23/4.8 | 112 | 12/33 |
| 3007 | polymerase (DNA directed), delta 1, catalytic subunit (Pold1) | gi|11067381 | 124.95/7.2 | 59 | 11/35 |
| 4011 | Hsc70-ps1 (Hspa8) | gi|56385 P63018 | 71.11/5.4 | 60 | 6/13 |
| 4502 | Pkm2 protein (Pkm2) | P11980 | 57.61/8.0 | 52 | 6/17 |
| 5002 | S100 calcium binding protein A11 (S100a11) | gi|51854249 Q6B345 | 11.23/5.6 | 67 | 5/8 |
| 6105 | Nucleoside diphosphate kinase B (Nme2) | gi|55926145 P19804 | 17.39/6.92 | 83 | 8/20 |
| 6106 | Similar to ribosomal protein S12 (Rps12) | gi|34854382 P63324 | 14.38/7.0 | 53 | 4/9 |
| 7101 | Actin, cytoplasmic 1 (β-actin) | P60711 | 42.05/5.3 | 52 | 4/8 |
| 7106 | Eukaryotic translation elongation factor 2 (EEf-2) | gi|8393296 P05197 | 96.12/6.41 | 56 | 8/21 |
| 7318 | Peroxiredoxin 4 (Prdx4) | gi|37590233 Q9Z0V5 | 31.22/6.2 | 161 | 13/27 |
| 8408 | Gapdh protein (Gapdh) | gi|37590767 P04797 | 36.09/8.1 | 162 | 18/29 |
| 8415 | Fructose-bisphosphate aldolase A (Aldoa) | gi|202837 P05065 | 39.65/8.4 | 52 | 7/20 |

Details of the experimental procedures are given in Materials and Methods. Positive control drug: Diltiazem hydrochloride.
